# Spillover benefit of improved access to healthcare on reducing worry about housing and meal affordability

**DOI:** 10.1186/s12939-018-0877-y

**Published:** 2018-11-22

**Authors:** Shiho Kino, Koryu Sato, Ichiro Kawachi

**Affiliations:** Department of Social and Behavioral Sciences, Harvard. T.H. Chan School of Public Health, 677 Huntington Avenue, Boston, MA 02115 USA

**Keywords:** Patient protection affordable care act, Health insurance, House stability, Food security, Causal inference

## Abstract

**Background:**

The Affordable Care Act Medicaid expansion improved access to health insurance among low-income populations. We sought to examine the spillover benefits of the ACA Medicaid expansion on ability to afford rent/mortgage and purchase of nutritious meals.

**Methods:**

Using data from the Behavioral Risk Factor Surveillance System (BRFSS) we analyzed individuals aged 18–64 years residing in 12 U.S. states (including five ACA Medicaid expansion states) in 2015. Our treatment of interest was access to health insurance, instrumented by the ACA Medicaid expansion. Our outcome variables were: worry or stress about having sufficient money to pay the rent or mortgage and to purchase nutritious meals. We conducted a two-stage least squares instrumental variables regression.

**Results:**

A 10%-point increase in the proportion of those who obtained health insurance following the ACA Medicaid expansion reduced the probability of being worried and stressed related to purchasing nutritious meals by 7.2% points (95% CI: 1.3–13.2) as well as paying the rent or mortgage by 8.6% points (95% CI: 2.5–14.7) among people living below 138% of the federal poverty level (FPL). The ACA Medicaid expansion was not associated with access to health insurance among those living over 138% of FPL, and obtaining health insurance did not influence stress or worry in relation to affording rent/mortgage or meals in this income group.

**Conclusions:**

Improved access to health insurance contributed to reducing worry and stress associated with paying rent/mortgage or purchasing meals among low-income people. Expanding health insurance access may have contributed to increasing the disposable income of low income groups.

## Background

Catastrophic medical expenditure is the leading cause of bankruptcy in the United States [1]. In fact, 62.1% of all bankruptcies in 2007 in the United States were medical reasons [2]. Under the Affordable Care Act (ACA), Medicaid eligibility was expanded to low-income adults with incomes below 138% of the federal poverty level (FPL) in 2014 in several states [3]. As a result, the ACA legislation succeeded in expanding insurance coverage [4–6] and reduced the probability of such an event of medical bankruptcy [7]. On the top of that, expanding access to health care can also be viewed as a “social determinant of health” because of its spillover (or collateral) benefits. For example, access to health insurance contributes to health not only directly by providing people with preventive care and treatment [8], but also indirectly by reducing out-of-pocket medical expenditures, and alleviating the stress or worry associated with being uninsured [6, 9–11].

Socioeconomic status is also one of the crucial determinants of health. Not only material disadvantage but also psychological distress related to financial situation are related to health [12, 13]. Tucker-Seeley et al. reported that financial hardship, which was measured by financial situation at the end of the month (viz., not enough money vs enough to make ends meet or some money left over), was associated with poor self-reported health [14]. Furthermore, other studies reported that financial hardships - such as difficulty in paying bills, ongoing financial strain, food insecurity and taking less medication due to the cost - were also related to self-rated health and even mortality [15, 16].

Various means-tested programs are currently offered in the United States to relieve the needs of low income families – e.g. Temporary Assistance for Needy Families, Supplemental Social Security Income, Section 8 housing vouchers, and the Supplemental Nutrition Assistance Program. These programs provide a safety net for families suffering from financial hardship by offering assistance in the form of cash, food, and housing. These programs may also have an indirect health benefit by relieving the psychological worry about being able to afford the basic necessities of life. In the United States, Medicaid is also a means-tested program to provide health insurance to low income families. Although the primary purpose of Medicaid is to enable poor families to access health care, a potentially overlooked health benefit of the program is to relieve psychological worry stemming from medical expenses. Indeed catastrophic medical expenses remains one of the chief reasons for personal bankruptcies in the U.S. [1]. Hence the passage of the Affordable Care Act provides a “natural experiment” to test the thesis that expanding health insurance access can have collateral health benefits in the form of reduced psychological worry about affording basic necessities.

Although there is obviously a debate that mandating the purchase of health insurance can reduce consumption budgets among certain people (i.e. working-poor population) [17], the freed coverage of medical expenses for those living under 138% of FPL could reduce out-of-pocket expenditures, thereby leaving more money for other necessities. Thus, from the viewpoint of basic theories of economics, reduction in costs of health care among people living below 138% of FPL would relax one’s budget constraint and increase consumption of other goods [18].

A previous study in Taiwan reported that free health care as a result of National Health Insurance introduced in 1995 resulted in an increase in overall non-medical spending and spending on housing-related expenses (i.e. rent and water bills) as well as a decrease in household medical expenditure [19]. In addition, even though the results were not statistically significant, they reported an increase in expenditure on transportation and communication, fuel and electricity bills, furniture and household appliances, household maintenance, tobacco and miscellaneous. On the other hand, they also reported a statistically significant decrease in education expenses, travel, entertainment and cultural activities, personal wardrobe and beverage, as well as a non-statistical decrease in food consumption [19]. Furthermore, Allen et al. examined the spillover effect of the ACA Medicaid expansion and reported the association between the expansion and a reduction in the number of loans, the number of unique borrowers and the amount of pay day loan debt [20]. Some other recent studies have also reported benefits of the ACA Medicaid expansion such as reducing personal bankruptcy, the amount of medical debt and the incidence of unpaid medical bills and improving credit scores [21–23]. Thus, the ACA Medicaid expansion may have relieved financial pressure on low income families suffering from the burden of medical expenses, and thereby improved their burden of financial worry, especially regarding their ability to afford other necessities of life such as housing and meals. Health insurance is thus hypothesized to have a spillover benefit on health that is independent of the sense of psychological security afforded by the knowledge that one’s health care needs are taken care of. However, to our knowledge, there have not been studies that examined the spillover effect of the ACA legislation on psychological aspects about affordability of basic necessities among low-income people.

This relationship between health insurance and household consumption is endogenous. For example, unhealthy people would be more likely to buy health insurance in order to limit their out-of-pocket medical expenditures. Conversely, healthy people would be more reluctant to voluntarily buy insurance (thereby reducing their disposable budget). There are also other unobserved confounders underlying the association between health insurance purchase and health, for insurance, more risk averse individuals are likely to buy health insurance regardless of their health status. Also, risk averse individuals might also be more prudent in their consumption of other items as avoiding getting into too much debt by purchasing a house that they cannot afford. Hence it is difficult to attribute causality to a correlation between insurance status and household consumption.

The Medicaid expansion, however, was not still implemented in 18 states in May 2018 because a 2012 U.S. Supreme Court decision made the expansion optional for states. The ACA legislation therefore provides a natural policy experiment to examine this question. In addition, the relationship between implementation of the Medicaid expansion and affordability of rent or meal is exogenous, i.e. the ACA Medicaid expansion does not directly influence stress or worry related to affordability of other necessities, but only via freed health insurance. This situation makes the instrument variables (IV) approach possible [24].

We, therefore, sought to investigate if obtaining free health insurance stemming from Medicaid expansion under ACA helped to reduce worries and stress related to paying rent or mortgage and buying nutritious meal among low-income Americans.

## Methods

### Data source and study sample

We used data from the Behavioral Risk Factor Surveillance System (BRFSS) in 2015. The BRFSS includes more than 400,000 individuals aged 18 years and older residing in the 50 states, the District of Columbia and three U.S. territories each year. The data are collected every year by telephone interview to monitor state trends in health-related risk behaviors, chronic health conditions, and use of preventive services [25].

In this study, we included 12 states that included the question on their survey about stress/worry related to paying rent/mortgage and buying nutritious meals in 2015: five ACA expansion states (Arkansas, Delaware, District of Columbia, Minnesota, and Rhode Island) and seven ACA non-expansion states (Alabama, Georgia, Louisiana, Mississippi, Missouri, Tennessee, and Utah).

We restricted the sample to respondents aged below 65 years, i.e. excluding Americans who are covered by Medicare. We assigned each respondent to a household income level based on the percentage of the federal poverty level (FPL) using the upper threshold of each income category and the reported household size, following the procedure described by Simon et al. [4].

### Outcome variables

We selected two outcome variables related to worry or stress of consumption behaviors, namely paying rent/mortgage and buying nutritious meals. The questions asked: How often in the past 12 months you were worried or stressed about having enough money to pay the rent/mortgage or to buy nutritious meals. The possible response options for these questions were [1] always, [2] usually, [3] sometimes, [4] rarely, and [5] never. We dichotomized the responses into 2 groups; a stressed group coded “1” including [1] always, [2] usually and [3] sometimes, and a not-stressed group coded “0” including [4] rarely and [5] never. The answer of don’t know/not sure, not applicable or refused was considered as missing.

### Covariate variables

We controlled for age, sex, race/ethnicity, education, household income, employment status, marital status, self-reported general health status, household size, living with children, as well as state-level characteristics, namely percentages of the state population uninsured, the unemployment rate, poverty rate, white population rate, education rate (those graduated high school or higher), foreign-born rate, and proportion of people aged over 65. All state-level data were obtained from the datasets of American Community Survey in 2015 [26], but the data on the unemployment rate was obtained from the Bureau of Labor Statistics in 2015 [27].

### Statistical analysis

Weighted percentages for all the variables of the sample population were calculated using the BRFSS weights to adjust for nonresponse and to make the total number of cases to approximate population estimates for each geographic region, controlling for age group by gender, race or ethnicity, education, marital status, tenure, gender by race or ethnicity, age group by race or ethnicity, and phone ownership [28].

We conducted the instrumental variables (IV) analysis with a two-stage least squares (2SLS) to estimate the causal effect of an exposure on an outcome regardless of the existence of unmeasured confounders in a natural experiment of policy changes [29, 30]. The application of a 2SLS model, equivalent to a linear probability model (LPM) with instrumental variables (IV), to binary outcomes has been previously described [31, 32]. We treated residence in an expansion state as the instrument and hypothesized that the ACA Medicaid expansion improved access to health insurance (stage 1), and obtaining health insurance in turn reduced stress or worry related to paying rent/mortgage or buying nutritious meals (stage 2). Our exclusion restriction assumes that ability to pay for rent/mortgage and buy meal is not directly influenced by the Medicaid expansion. We stratified the samples into two groups by FPL; 138% or less and over 138% to confirm our assumptions on the IV analysis. The subgroup of households with incomes below 138% of FPL were eligible for Medicaid expansion, whereas the subgroup of incomes over 138% of FPL were not. There is no difference among those with over 138% of FPL between expansion and non-expansion states in the subsidized coverage by Marketplace, cost-sharing reduction and a penalty for non-insured people. In the first stage, the model estimated the association between the ACA expansion and obtaining health insurance using linear regressions controlling for all the covariates to compare residents in states which implemented the legislation and those in states which did not. In the second stage, we examined the relationship between the instrumented probability of obtaining health insurance and stress/worry related to paying rent or buying nutritious meal. The analyses were conducted in each FPL group in order to compare between the population targeted Medicaid expansion and non-targeted population. All analyses were conducted by STATA 14 [33].

As a sensitivity analysis, we included an interaction term between self-rated health status and health insurance because healthy low-income people might not get benefit directly from having health insurance.

## Results

Table 1 presents the demographics of the samples. The group of 138% or less of FPL included more women, single adults, younger people, non-white people, less educated and uninsured people, more individuals with a self-reported poor health condition, unemployment status, and living with children aged 18 years or younger than the group with above 138% of FPL. Regarding state-level factors, non-expansion states had higher mean percentages of uninsured-rate and poverty-rate while expansion states had a higher mean percentage of foreign-born residents (Appendix).

**Table 1:** Characteristics of the sample

	138% of FPL or less	More than 138% of FPL
	Weighted %	Weighted %
Sex		
Men	36.37	45.66
Women	63.63	54.34
Age		
18–24	11.57	5.83
25–34	18.66	14.94
35–44	19.05	18.90
45–54	22.44	25.75
55–64	28.28	34.58
Marital status		
Single	66.95	32.98
Married	33.05	67.02
Race		
White (non-Hispanic)	55.65	81.52
Black	28.93	11.38
Hispanic	9.32	3.16
Multiracial	4.29	2.77
Other	1.81	1.18
Income		
Less than $10,000	27.56	0
$10,000 to less than $15,000	23.02	0
$15,000 to less than $20,000	23.70	2.36
$20,000 to less than $25,000	17.69	5.24
$25,000 to less than $35,000	6.00	9.76
$35,000 to less than $50,000	2.03	15.88
$50,000 to less than $75,000	0	20.62
$75,000 or more	0	46.14
Education		
Did not graduate High School	18.09	3.03
Graduated High School	39.64	22.73
Attend College/Technical School	29.47	29.29
Graduated from College/Technical School	12.81	44.95
Health insurance		
Uninsured	24.07	6.27
Insured	75.93	93.73
General health		
Poor	37.11	11.11
Good	62.89	88.89
Employment status		
Unemployed	61.87	24.11
Employed	38.13	75.89
Having children (18 or less)		
No	52.65	61.62
Yes	47.35	38.38
Household size (mean)	3.21	2.82

Table 2 shows the results of 2SLS analysis examining the influence of improved access to health insurance (stemming from ACA Medicaid expansion) on stress/worry related to purchase of nutritious meals in each FPL group. In the first stage regression, among people with 138% or less of FPL, we found that the people living in Medicaid expansion states were significantly more likely to have obtained health insurance (F-statistic of 20.47), suggesting that the ACA Medicaid expansion is a strong instrument. In the second stage, we found that a 10%-point increase in having health insurance reduced the likelihood of stress or worries related to buying nutritious meals by 7.2% (95%CI: 1.3, 13.2). On the other hand, living in a Medicaid expansion state was not associated with higher insurance rates among those with incomes above 138% of FPL. Also, we found that obtaining health insurance was not associated with stress or worry related to buying nutritious meal among those with incomes above 138% of FPL (Coef. -1.83; 95%CI: -4.77, 1.11).

**Table 2:** Results from instrumental variable regression analysis examining the influence of improved access to health insurance stemming from ACA Medicaid expansion on stress or worry to buy nutritious meal

	138% or less of FPL		More than 138% of FPL	
	[*N* = 7841]		[*N* = 31,705]	
	Coef. (95% CI)	*p*-value	Coef. (95% CI)	p-value
*2nd-stage regression*				
Insurance (ref. uninsured)	−0.72 (−1.32, −0.13)	0.017	−1.83 (−4.77, 1.11)	0.222
Age	0.01 (−0.01, 0.01)	0.068	−0.01 (−0.02, 0.02)	0.955
Sex (ref. male)	0.14 (0.10, 0.18)	< 0.001	0.08 (0.03, 0.14)	0.005
Marital status (ref. single)				
Married	−0.02 (− 0.05, 0.01)	0.261	0.01 (− 0.04, 0.05)	0.834
Race (ref. White - non-Hispanic)				
Black	−0.09 (− 0.12, − 0.05)	< 0.001	0.02 (− 0.02, 0.05)	0.362
Hispanic	−0.20 (− 0.35, − 0.05)	0.008	− 0.12 (− 0.30, 0.06)	0.179
Multiracial	0.03 (− 0.04, 0.09)	0.406	0.02 (− 0.04, 0.07)	0.554
Other	− 0.01 (− 0.09, 0.08)	0.934	0.04 (− 0.03, 0.10)	0.263
Income				
Less than$10,000	(ref)			
$10,000 to less than $15,000	0.05 (0.01, 0.10)	0.041		
$15,000 to less than $20,000	−0.01 (− 0.05, 0.04)	0.758	0.18 (− 0.17, 0.53)	0.322
$20,000 to less than $25,000	−0.01 (− 0.07, 0.05)	0.700	0.16 (− 0.17, 0.50)	0.343
$25,000 to less than $35,000	0.01 (−0.12, 0.12)	0.971	0.12 (−0.12, 0.36)	0.316
$35,000 to less than $50,000	0.06 (−0.12, 0.24)	0.530	0.06 (− 0.10, 0.23)	0.455
$50,000 to less than $75,000			0.06 (0.01, 0.11)	0.028
$75,000 or more			(ref)	
Education (ref. Did not graduate High School)				
Graduated High School	0.03 (−0.02, 0.07)	0.296	0.13 (−0.18, 0.45)	0.414
Attend College/Technical School	0.05 (−0.01, 0.11)	0.101	0.19 (−0.20, 0.59)	0.339
Graduated from College/Technical School	−0.01 (− 0.08, 0.06)	0.718	0.17 (− 0.25, 0.60)	0.423
Self-rated general health	−0.19 (− 0.22, − 0.16)	< 0.001	− 0.20 (− 0.27, − 0.14)	< 0.001
Employment status	− 0.10 (− 0.16, − 0.04)	0.001	0.01 (− 0.02, 0.03)	0.627
Having children	0.05 (0.02, 0.08)	0.004	0.05 (0.01, 0.10)	0.036
Household size	−0.01 (− 0.02, − 0.01)	0.027	0.01 (− 0.01, 0.02)	0.146
State-level uninsured-rate	− 0.03 (− 0.04, − 0.01)	< 0.001	−0.01 (− 0.02, − 0.01)	0.008
State-level unemployment-rate	−0.02 (− 0.07, 0.02)	0.342	0.02 (− 0.01, 0.04)	0.098
State-level poverty-rate	0.01 (−0.01, 0.04)	0.291	− 0.01 (− 0.02, 0.01)	0.148
State-level white people-rate	0.01 (−0.01, 0.01)	0.257	0.01 (−0.01, 0.01)	0.551
State-level education-rate	−0.01 (− 0.04, 0.02)	0.424	− 0.01 (− 0.03, 0.01)	0.249
State-level foreign born-rate	0.01 (− 0.01, 0.02)	0.457	− 0.01 (− 0.01, 0.01)	0.733
State-level rate of people aged 65+	− 0.01 (− 0.01, 0.01)	0.893	− 0.01 (− 0.01, 0.01)	0.366
*1st-stage regression*				
ACA	0.13 (0.07, 0.19)	< 0.001	0.01 (−0.01, 0.03)	0.123
F-statistic	20.47		2.75	

Table 3 shows the results of the 2SLS analysis with stress or worry related to paying rent or mortgage as the outcome. The first stage F statistic was 21.28 and ACA was significantly associated with obtaining health insurance (Coef. 0.14; 95%CI: 0.08, 0.20) among eligible households. In the second stage, we found that a 10%-point increase in access to health insurance was associated with reduced worry and stress related to affording rent/mortgage among households by 8.6%-points with incomes 138% or less of FPL (Coef. -0.86; 95%CI: -1.47, − 0.25). On the other hand, there was no significant association between obtaining health insurance and stress or worry related to paying rent or mortgage among those with incomes above 138% of FPL.

**Table 3:** Results from instrumental variable regression analysis examining the influence of improved access to health insurance stemming from ACA Medicaid expansion on stress or worry to pay rent or mortgage

	138% of FPL or less		More than 138% of FPL	
	[*N* = 7013]		[*N* = 30,438]	
	Coef. (95% CI)	p-value	Coef. (95% CI)	p-value
*2nd-stage regression*				
Insurance (ref. uninsured)	−0.86 (−1.47, − 0.25)	0.006	−3.94 (−11.04, 3.15)	0.276
Age	0.01 (0.01, 0.02)	0.043	0.02 (−0.03, 0.07)	0.484
Sex (ref. male)	0.09 (0.06, 0.13)	< 0.001	0.10 (−0.03, 0.23)	0.139
Marital status (ref. single)				
Married	−0.03 (− 0.06, 0.01)	0.200	0.02 (− 0.06, 0.10)	0.569
Race (ref. White - non-Hispanic)				
Black	−0.02 (− 0.05, 0.02)	0.364	0.04 (− 0.03, 0.10)	0.294
Hispanic	−0.27 (− 0.43, − 0.11)	0.001	− 0.24 (− 0.65, 0.17)	0.250
Multiracial	−0.01 (− 0.08, 0.06)	0.791	− 0.02 (− 0.12, 0.09)	0.765
Other	0.01 (− 0.10, 0.11)	0.957	0.04 (− 0.07, 0.16)	0.480
Income				
Less than $10,000	(ref)			
$10,000 to less than $15,000	0.02 (−0.03, 0.07)	0.501		
$15,000 to less than $20,000	−0.01 (− 0.05, 0.04)	0.912	− 0.02 (− 0.85, 0.81)	0.966
$20,000 to less than $25,000	− 0.02 (− 0.09, 0.05)	0.549	− 0.05 (− 0.87, 0.77)	0.902
$25,000 to less than $35,000	− 0.04 (− 0.16, 0.08)	0.500	0.01 (− 0.56, 0.56)	0.992
$35,000 to less than $50,000	−0.01 (− 0.19, 0.17)	0.877	0.01 (− 0.40, 0.41)	0.987
$50,000 to less than $75,000			0.06 (− 0.06, 0.18)	0.304
$75,000 or more			(ref)	
Education (ref. Did not graduate High School)				
Graduated High School	0.02 (−0.03, 0.07)	0.364	0.38 (−0.36, 1.12)	0.314
Attend College/Technical School	0.04 (−0.02, 0.10)	0.219	0.48 (−0.44, 1.41)	0.306
Graduated from College/Technical School	0.01 (−0.06, 0.08)	0.825	0.47 (−0.53, 1.47)	0.356
Self-rated general health	−0.15 (− 0.19, − 0.12)	< 0.001	− 0.24 (− 0.37, − 0.10)	< 0.001
Employment status	− 0.05 (− 0.11, 0.01)	0.093	0.05 (0.01, 0.08)	0.015
Having children	0.08 (0.05, 0.12)	< 0.001	0.09 (− 0.02, 0.19)	0.106
Household size	−0.01 (− 0.01, 0.01)	0.828	0.01 (− 0.02, 0.04)	0.564
State-level uninsured-rate	−0.03 (− 0.04, − 0.01)	< 0.001	−0.02 (− 0.04, − 0.01)	0.042
State-level unemployment-rate	−0.04 (− 0.08, 0.01)	0.153	0.03 (− 0.02, 0.08)	0.205
State-level poverty-rate	0.02 (− 0.01, 0.05)	0.211	−0.01 (− 0.03, 0.01)	0.392
State-level white people-rate	0.01 (− 0.01, 0.01)	0.588	− 0.01 (− 0.01, 0.01)	0.946
State-level education-rate	−0.01 (− 0.04, 0.02)	0.498	− 0.01 (− 0.04, 0.03)	0.763
State-level foreign born-rate	0.01 (− 0.01, 0.03)	0.157	− 0.01 (− 0.01, 0.01)	0.640
State-level rate of people aged 65+	0.01 (− 0.01, 0.02)	0.300	0.01 (− 0.01, 0.02)	0.615
*1st-stage regression*				
ACA	0.14 (0.08, 0.20)	<0.001	0.01 (−0.01, 0.03)	0.240
F-statistic	21.28		1.61	

Among people with income below 138% of FPL, stress or worry related to affording meals and rent or mortgage was less pronounced among people with better self-reported health status (Coef. -0.19; 95%CI: -0.22, − 0.16, Coef. -0.15; 95%CI: -0.19, − 0.12, respectively). In addition, a sensitivity analysis did not show statistically significant interactions between self-rated health status and having health insurance for either outcome.

## Discussion

The ACA originally designed to lower the uninsured rate in the United States [3], also appears to have resulted in a positive spillover effect by reducing stress and worry associated with affording nutritious meals and paying the rent or mortgage among low-income people. Our study supports the notion that expanding access to health insurance helps to lower consumption budget constraints among low-income households [17].

Our findings are consistent with a previous study in California showing that the Medicaid expansion reduced loans and payday borrowing [20]. In addition, our findings are partially in line with the natural policy experiment related to the introduction of the National Health Insurance scheme in Taiwan in 1995, which reported that free health insurance was associated with an increase in expenditure on water and rent [19]. On the other hand, Sheu and Lu (2014) reported no effect on spending on food, whereas we found a positive effect on reducing stress or worry related to buying nutritious meals. The difference between their study and our study could be explained firstly by the fact that the ACA Medicaid expansion targeted low-income families whereas the Taiwan policy change applied to the whole population. Food insecurity is more likely to affect low-income populations. Secondly, our study examined stress or worry related to affordability while the Taiwan study examined actual spending shares. Thus, our study outcome was focused on the psychological aspect of food security, while the Taiwan study examined actual consumption behavior. The psychological aspects were measured in our study by how stressed or worried people felt regardless of their actual spending on housing and food. Financial hardship is both an objective state (as reflected by changes in consumption) as well as a subjective state (perceptions of security). Both aspects matter for health.

Unmet needs for adequate nutrition, housing and healthcare are each determinant of health outcomes [34]. For example, housing costs can be a significant source of financial strain [35]. Financial strain associated with housing is in turn correlated with worse health status and poor health behaviors (e.g., poor self-rated health, hypertension, arthritis, cost-related healthcare nonadherence, and cost-related prescription nonadherence) [35, 36]. In turn, poor health resulting from financial strain may result in excess health expenditures, creating further pressure on household budgets. Expanding access to health insurance may be an effective way to break this cycle.

Furthermore, we found that stress or worry related to affordability of nutritious meals and rent/mortgage was associated with being female, living with children, and being unemployed. These are the groups most likely to benefit from ACA Medicaid expansion. Our study also found that low-income people with better self-reported health status were less likely to report stress or worry related to affording meals and rent or mortgage. This might be because people with better health have less reason to be worried by future medical costs. Hence, they are less likely to be impacted by expansion of Medicaid coverage.

Some limitations of our study should be noted. Due to the fact that the BRFSS is a telephone interview survey, the most disadvantaged segments of the population may have been missed. However, the survey included cellular telephone respondents as well as landline telephone respondents, and adjusted for the overlapping sample frames using the weights. In addition, the response rate in the BRFSS is slightly less than 50%, although this is still significantly higher compared to other telephone surveys. We used BRFSS survey weights to improve the representativeness of the population.

## Conclusions

In conclusion, improved access to health insurance contributes to reducing worry and stress associated with affording nutritious meals as well as paying the rent or mortgage among low-income people. Expanding health insurance access may have contributed to increasing the disposable income of low income groups.

## Appendix

**Table 4:** Mean of state-level variables in ACA Medicaid expansion states and non-expansion states

	Expansion states (%)	Non-expansion states (%)
Uninsured-rate	5.28	10.53
Unemployment-rate	5.30	5.57
Poverty-rate	14.58	17.13
White people-rate	70.24	70.93
Education-rate	88.92	86.51
Foreign born-rate	10.00	5.30
Rate of people aged 65+	27.28	26.99

## Abbreviations

ACA: Accordable Care Act
BRFSS: Behavioral Risk Factor Surveillance System
FPL: federal poverty level
IV: instrument variables
LPM: linear probability model
